# Sensitive detection of *Treponema pallidum* DNA from the whole blood of patients with syphilis by the nested PCR assay

**DOI:** 10.1038/s41426-018-0085-2

**Published:** 2018-05-09

**Authors:** Cuini Wang, Yuanyuan Cheng, Biao Liu, Yuanyuan Wang, Weiming Gong, Yihong Qian, Zhifang Guan, Haikong Lu, Xin Gu, Mei Shi, Pingyu Zhou

**Affiliations:** 1 STD Institute, Shanghai Skin Disease Hospital, Shanghai, China; 2 School of Laboratory Medicine, Wenzhou Medical University, Wenzhou, China; 3 Shanghai Skin Disease Hospital, Clinical School of Anhui Medical University, Shanghai, China; 4 Present Address: Clinical Laboratory Center, Hunan Children’s Hospital, Changsha, China

## Abstract

The aim of this work was to investigate the application of the nested PCR assay for the detection of *Treponema pallidum* (TP) DNA from the blood of patients with different stages of syphilis. In this study, a nested PCR method targeting the *Tpp47* and *polA* genes (*Tpp47*-Tp-PCR and *polA*-Tp-PCR) was developed to detect TP-DNA in whole blood samples collected from 262 patients with different stages of syphilis (84 primary syphilis, 97 secondary syphilis, and 81 latent syphilis patients). The PCR assay detected *T. pallidum* DNA in 53.6% and 62.9% of the patients with primary and secondary syphilis, respectively, which was much higher than the detection levels in patients with latent syphilis (7.4%) (both *p* < 0.001). For primary syphilis, a low RPR (0–16) was correlated with a higher detection rate of TP-DNA, whereas for secondary syphilis, the higher detection rate of blood TP-DNA was correlated with higher blood RPR titers (at or beyond 32). For latent syphilis, TP-DNA was only detectable by PCR in the early phase of the latent infection. Thus, blood RPR titers were correlated with the blood *T. pallidum* burden, but the correlations varied with primary and secondary syphilis. The results indicate that nested PCR is a sensitive method for detecting blood TP-DNA and is especially useful for detecting early syphilis including primary syphilis and secondary syphilis. The findings also suggest that the PCR assay may be used to complement other methods to enhance the diagnosis of syphilis.

## Introduction

The incidence of syphilis has experienced a sharp increase worldwide over the last several decades^1^. In China, cases of syphilis showed a markedly ascending trend from 2004 to 2013, with an annual percentage increase of 16.3% (95% confidence interval (CI) 13.8 to 18.8)^2^. In 2016, 438,199 new syphilis cases were reported^3^.

*Treponema pallidum* is the etiological agent of syphilis and it is known that *T. pallidum* can disseminate to the central nervous system within days after exposure, leading to severe and irreversible symptomatic neurosyphilis if left untreated^4^. Previous studies have suggested that certain strain types of *T. pallidum* were associated with neurosyphilis^5,6^. However, few studies have assessed blood and cerebrospinal fluid (CSF) samples due to the difficulty in extracting and detecting low amounts of target DNA, and molecular typing of *T. pallidum* has mostly been done with specimens from moist skin lesions^5^. Therefore, data are still limited about *T. pallidum* in the blood and CSF, and there is still a lack of concrete evidence for the higher-risk strain types associated with neurosyphilis. If strain types can be determined directly from blood specimens, a large number of clinical samples may be analyzed, which will help identify the preferential strain types associated with neurosyphilis. In addition, the improved detection of *T. pallidum* will help in the early and rapid diagnosis of syphilis. Hence, an efficient technology to enhance the detection of *T. pallidum* DNA in the blood should be very useful for understanding the epidemiology and pathogenesis of syphilis.

At present, several PCR methods have been developed for the detection of *T. pallidum*^7–13^. However, in the case of single-step PCR using clinical specimens, the low quantity of DNA present in the specimens will greatly affect the sensitivity. To enhance the detection sensitivity, nested PCR was therefore used in this study for the detection of *T. pallidum* in whole blood samples from patients with syphilis. The correlation between the presence of *T. pallidum* DNA with blood rapid plasma reagin (RPR) titer and different stages of the disease was comprehensively evaluated.

## Results

### Nested PCR assays (*Tpp47*-Tp-PCR and *polA*-Tp-PCR) were used to detect *T. pallidum* DNA in blood samples from all patients

**Fig. 1:**
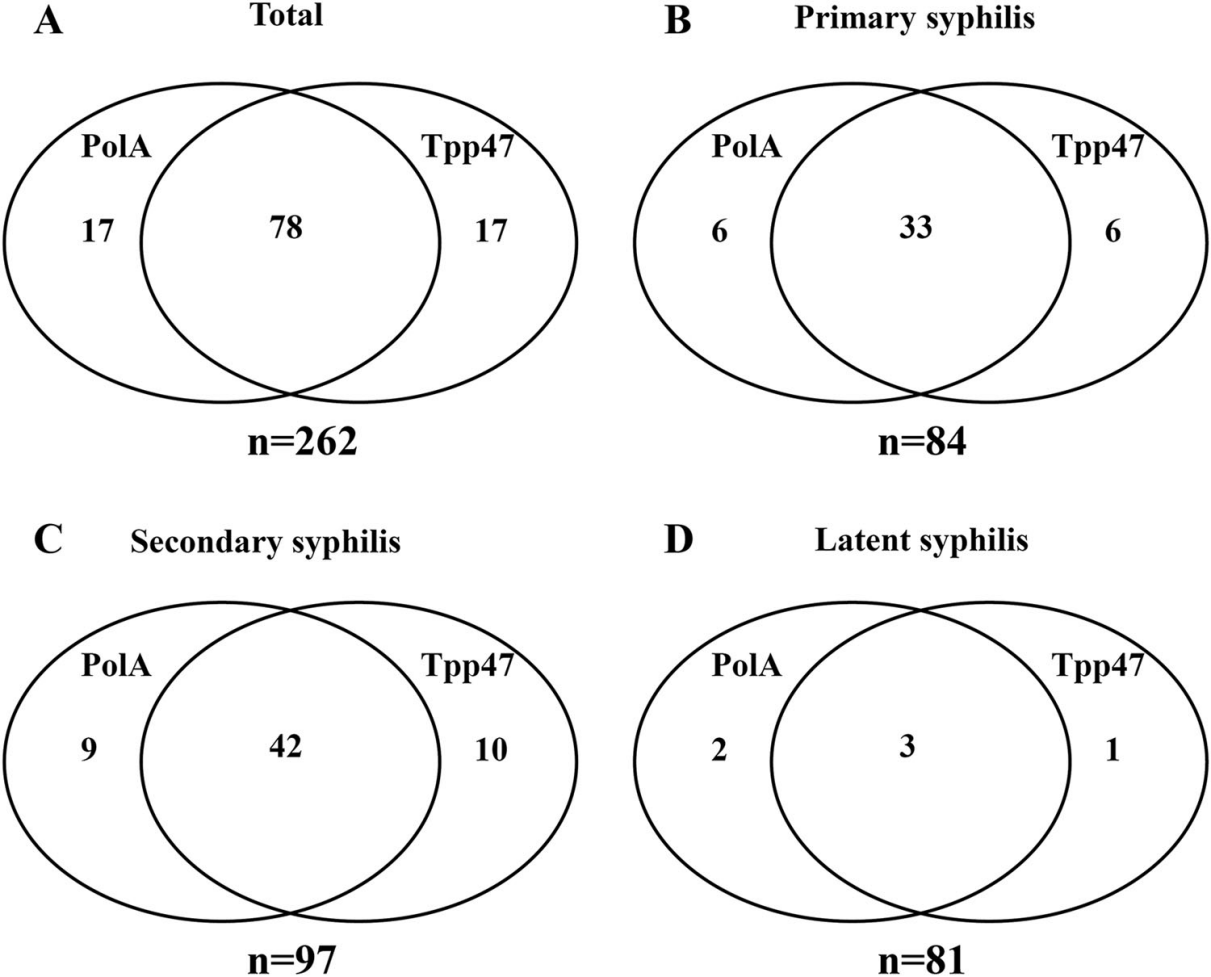
The detection rate of *T. pallidum* DNA in blood samples from all patients by *Tpp47*-Tp-PCR and *polA*-Tp-PCR. **a** The positive specimens of *polA*-Tp-PCR and/or *Tpp47*-Tp-PCR among all patients (*n* = 262). **b** The positive specimens of *polA*-Tp-PCR and/or *Tpp47*-Tp-PCR in the primary syphilis patients (*n* = 84). **c** The positive specimens of *polA*-Tp-PCR and/or *Tpp47*-Tp-PCR in the secondary syphilis patients (*n* = 97). **d** The positive specimens of *polA*-Tp-PCR and/or *Tpp47*-Tp-PCR in the latent syphilis patients (*n* = 81)

In total, 262 patients were included in this study. Among them, 84 (32.1%) patients were with primary syphilis, 97 (37.0%) with secondary syphilis, and 81 (30.9%) with latent syphilis (Table 1). The PCR detection targeted *polA* and *Tpp47*. As shown in Fig. 1a, 95 (36.3%) specimens were* polA*-Tp-PCR positive and 95 (36.3%) were *Tpp47*-Tp-PCR positive. In total, 112 (42.7%) patients were positive with *Tpp47*-Tp-PCR and/or* polA*-Tp-PCR. We obtained 78 concordant-positive samples and thus 150 concordant-negative Tp-PCR results; 17 specimens were negative for *polA*-Tp-PCR but positive for *Tpp47*-Tp-PCR, whereas 17 were negative for *Tpp47*-Tp-PCR but positive for *polA*-Tp-PCR. There were no significant differences between the two target genes (*p* = 1.000) and the *κ*-coefficient was 0.719.

**Table 1:** Clinical characteristics of the 262 syphilis patients examined in this study

Variable	Primary syphilis	Secondary syphilis	Latent syphilis
Patient number, *n*	84	97	81
Age (median, IQR), years	50 (32–58)	35 (28–52)	44 (32–56)
Male gender, *n* (%)	75 (89.3)	71 (73.2)	44 (54.3)
Blood TPPA, *n* (%)	76 (90.5)	97 (100)	81 (100)
Blood RPR, *n* (%)	68 (81.0)	97 (100)	81 (100)
1/Serum RPR titer (median, IQR)	8 (1–64)	64 (32–128)	16 (8–64)
HIV infection, *n* (%)	5 (6.0)	15 (15.5)	2 (2.5)
CSF protein, g/l (median, IQR)	424 (298–524)*	303.5 (231.3–410)	340 (236.5–524)
CSF WBC, /μl	0 (0–4)*	4 (0–8)	1 (0–10)
CSF VDRL(+), *n* (%)	1 (5.9)*	9 (9.3)	16 (19.8)
CSF TPPA(+), *n* (%)	1 (5.9)*	30 (30.9)	35 (43.2)

IQR, interquartile range; RPR, rapid plasma reagin; TPPA, *T. palladium* particle agglutination; VDRL, venereal disease research laboratory; WBCs, white blood cells.

*The number of CSF sample was 17 (*n* = 17).

We also detected *polA* and *Tpp47* using single-step PCR among 95 *polA*-positive samples and 95 *Tpp47*-positive samples by nested PCR. We found that there were only 23.2% (22/95) polA- and 26.3% (25/95) *Tpp47*-positive samples. The grayscale of DNA fragments of *Tpp47* and *polA* were very weak compared with those of the nested PCRs (Supplementary Figure 1).

### The detection rate of *T. pallidum* DNA in the blood from patients with different clinical stages of syphilis

As shown in Fig. 1b, 45 (53.6%) specimens from 84 patients with primary syphilis were positive for *Tpp47*-Tp-PCR and/or *polA*-Tp-PCR, including 33 specimens positive for both *Tpp47*-Tp-PCR and *polA*-Tp-PCR, 6 specimens positive only for *Tpp47*-Tp-PCR, and 6 specimens positive only for* polA*-Tp-PCR.

For secondary syphilis, 42 (43.3%) specimens were positive for *polA*-Tp-PCR and *Tpp47*-Tp-PCR, 10 specimens were only positive for *Tpp47*-Tp-PCR, and 9 specimens were only positive for *polA*-Tp-PCR. In total, 61 (62.9%) specimens were positive for* polA*-Tp-PCR and/or *Tpp47*-Tp-PCR **(**Fig. 1c).

Of the 81 patients with latent syphilis, 5 (6.2%) specimens were positive for *polA*-Tp-PCR and 4 (4.9%) were positive for *Tpp47*-Tp-PCR. Among the positive specimens, 2 were only positive for *polA*-Tp-PCR and 1 was only positive for *Tpp47*-Tp-PCR. There were 6 (7.4%) specimens positive for *polA*-Tp-PCR and/or *Tpp47*-Tp-PCR in latent syphilis **(**Fig. 1d**)**.

In conclusion, the detection rate was the highest for secondary syphilis, but there was no significant difference in the detection rates between primary and secondary syphilis (*p* = 0.228). For latent syphilis, the detection rate was much lower compared with those of primary and secondary syphilis (both *p* < 0.001).

### The relationship between blood RPR titer and the presence of *T. pallidum* DNA in different stages of syphilis

The association between the blood RPR titer and the presence of *T. pallidum* DNA in different stages of syphilis is shown in Table 2. The positive rate of *T. pallidum* DNA was higher when the blood RPR titer was lower for primary syphilis. There were 53 primary syphilis patients with blood RPR titers ranging from 0 to 16, of whom 32 (60.4%) were positive with *polA*-Tp-PCR and/or *Tpp47*-Tp-PCR. For the 22 patients with blood RPR titers ranging from 32 to 64, 10 (45.5%) were positive for *polA*-Tp-PCR and/or *Tpp47*-Tp-PCR. There were only nine patients with blood RPR titers ≥ 128, of whom three (33.3%) were positive for *polA*-Tp-PCR and/or *Tpp47*-Tp-PCR. From the results, it is clear that the detection rate of TP-DNA was the highest (60.4%) when the blood RPR titer ranged from 0 to 16, as opposed to compared with those when the RPR titer ranged from 32 to 64 (45.5%) or when it was at or beyond 128 (33.3%); however, these differences did not reach statistical significance (*p* = 0.176 and 0.126, respectively). Interestingly, 8 of the 84 patients with primary syphilis were negative for blood RPR and *T. pallidum* particle agglutination assay (TPPA), but 5 (62.5%) of the 8 patients were blood TP-DNA positive. There were eight patients who were non-reactive with blood RPR but positive for blood TPPA; of these, two (25%) were Tp-DNA positive.

**Table 2:** The relationship between the blood RPR titer and the presence of *T. pallidum* DNA for all syphilis patients

	No. of patients with primary syphilis (*n* = 84)				No. of patients with secondary syphilis (*n* = 97)				No. of patients with latent syphilis (*n* = 81)			
RPR titer	Tested	*PolA*(+)	*Tpp47*(+)	*PolA*&*Tpp47*(+)	Tested	*PolA*(+)	*Tpp47*(+)	*PolA*&*Tpp47*(+)	Tested	*PolA*(+)	*Tpp47*(+)	*PolA*&*Tpp47*(+)
0–16	53	28 (52.8%)	26 (49.1%)	32 (60.4%)	8	1 (12.5%)	3 (37.5%)	3 (37.5%)	40	0	0	0
32–64	22	9 (40.9%)	10 (45.5%)	10 (45.5%)	49	28 (57.1%)	27 (55.1%)	33 (67.3%)	27	1 (3.7%)	2 (7.4%)	2 (7.4%)
≥ 128	9	2 (22.2%)	3 (33.3%)	3 (33.3%)	40	22 (55.0%)	22 (55.0%)	25 (62.5%)	14	4 (28.6%)	2 (14.3%)	4 (28.6%)

*PolA*(+), specimens positive for *polA*-Tp-PCR; RPR, rapid plasma reagin; *Tpp47*(+), specimens positive for *Tpp47*-Tp-PCR; *PolA*&*Tpp47*(+), specimens positive for* polA*-Tp-PCR and/or *Tpp47*-Tp-PCR.

In contrast to the situation with primary syphilis, the positive rate of *T. pallidum* DNA was higher for secondary syphilis when blood RPR titers were higher (Table 2). All patients with secondary syphilis were reactive to blood RPR and TPPA. There were eight patients with blood RPR titers ≤ 16, of whom three (37.5%) were positive for *polA*-Tp-PCR and/or *Tpp47*-Tp-PCR. When the blood RPR titer was in the range of 32 to 64, there were 49 patients of whom 33 (67.3%) were positive for *polA*-Tp-PCR and/or *Tpp47*-Tp-PCR. When the blood RPR titer was at or beyond 128, there were 40 patients of whom 25 (62.5%) were positive for *polA*-Tp-PCR and/or *Tpp47*-Tp-PCR. Thus, the detection rate of blood TP-DNA was higher when the blood RPR titer ranged from 32 to 64 (67.3%) or when it was at or beyond 128 (62.5%) than in patients with blood RPR titers ranging from 0 to 16 (37.5%); however, the differences were not statistically significant (*p* = 0.111 and 0.18, respectively).

There were only 6 positive specimens among 81 patients with latent syphilis. Fourteen patients had RPR titers at or beyond 128. Of these, four (28.6%) patients were positive for *polA*-Tp-PCR and/or *Tpp47*-Tp-PCR. In addition, there were 27 patients with RPR titers ranging from 32 to 64, 2 (7.4%) of whom were positive for *polA*-Tp-PCR and/or *Tpp47*-Tp-PCR. Both had blood RPR titers of 64. None of the patients were TP-DNA positive when the blood RPR titer ranged from 0 to 16. Among the six patients, three were early latent patients and three patients were of unknown duration.

## Discussion

The prompt diagnosis of early syphilis not only helps stop the transmission of the infection source but also prevents long-term consequences. However, in many circumstances, the diagnosis of early syphilis can be a challenge. For example *T. pallidum* could not be found at the site of bacterial infection; although it is very common clinically, its serum RPR/TPPA is negative. Therefore, a sensitive method for detecting *T. pallidum* is fundamental for the prompt diagnosis of early syphilis. In this study, the results showed that nested PCR might be a relatively sensitive method for detecting *T. pallidum* DNA in blood, especially in patients with primary and secondary syphilis. Our method was sensitive and the amount of target DNA fragments was low in the single-step PCR detection, indicating that the concentration of *T. pallidum* DNA was also low in the blood. When there were low amounts of templates in the samples, there was low sensitivity, instability, and poor repeatability using single-step PCR. Previous studies reported that, because Herpesvirus DNA was present in low copy numbers in abscess specimens, Herpesviral DNA could not be detected using primary PCR, and all abscess specimens required nested PCR for the detection of amplified DNA^14^. Our results confirmed that nested PCR provided a relatively sensitive method for detecting the pathogenic DNA in blood. To date, no specific genes have been recommended as a gold-standard target for TP-PCR. However, the *Tpp47* and *polA* genes are the most commonly used targets for PCR in previously reported studies^15^. *Tpp47* encodes a cytoplasmic membrane protein involved in cell wall synthesis and is partly specific for *T. pallidum* subsp. *pallidum*^16^. *polA* encodes DNA polymerase I involved in DNA repair and replication in most bacteria and shows a number of unique features in *T. pallidum* subsp. *pallidum*^17^. A recent study showed that Tp-PCR targeting the* Tpp47* and *polA* genes showed very good congruence and only four discordant results were observed between the two methods among 272 specimens from patients with sexually transmitted ulcers^18^. Our results also found good concordance between the two PCR targets (Fig. 1) and were consistent with the findings reported in previous studies.

This study showed that *T. pallidum* DNA could be amplified from whole blood samples in different stages of syphilis, which will help in the early diagnosis of syphilis. For spirochetemia in the secondary stage, the detection rate was the highest in secondary syphilis, reaching 62.9%. The detection of *T. pallidum* DNA for primary syphilis was 53.6% (45/84). Primary syphilis appears as painless ulcers at the site of *T. pallidum* contact. The bacteria mainly proliferate locally and spread into the blood at the same time. Unfortunately, the local bacteria, which are expected to be found easily under dark-field microscopic examination from genital ulcer swabs in patients with primary syphilis, can be easily destroyed under many circumstances. For example, topical antibiotics can destroy them, making the diagnosis of primary syphilis challenging when combined with a negative RPR and TPPA result. Our detection rate was higher than that previously reported. Grange et al.^13^ showed that the frequency of *T. pallidum* detection in whole blood using nested PCR was 13%. Quantitative PCR results from the study by Tipple et al.^11^ reported a 21.4% detection rate. On the one hand, subtle or atypical dermatological or mucosal lesions of syphilis can lead to misclassification of disease stages, and therefore primary and secondary syphilis may coexist. On the other hand, we tried our best to obtain reliable patient histories and none of our patients were treated with antibiotics before blood was collected in this study.

An interesting finding of this study is that *T. pallidum* DNA was detected in five patients with primary syphilis, who were non-reactive to both blood RPR and TPPA, indicating that *T. pallidum* can enter the blood shortly after infecting skin or mucosal surfaces. This finding can help physicians make an early diagnosis for those patients with atypical syphilis manifestations and negative serological responses. A previous study also showed that *T. pallidum* DNA could be detected in 3 (27%) of 11 individuals who were exposed to patients with syphilis and had a non-reactive blood RPR^19^. The entry of *T. pallidum* into the blood of experimentally infected animals occurs within 24 h of intratesticular inoculation^7^, but it is unknown whether this also occurs in humans. It is known that humoral antibodies against cardiolipin and treponemal antigen usually do not appear until 1–4 weeks after the development of a chancre^20^. Therefore, the interpretation of syphilis serology can be problematic for very early primary syphilis, because serological responses are delayed. Dark-field microscopy is very useful for new, moist primary lesions, but the detection sensitivity decreases as the lesions heal or are treated by local rinsing or antibiotic medications. This situation often requires patients to return for re-testing. Thus, *T. pallidum* DNA detection in blood provides not only an added value for the diagnosis of these cases of suspected primary syphilis but also gives evidence to facilitate proper and prompt treatment.

Another interesting finding of this study was that blood *T. pallidum* DNA was more commonly detected in patients with low serum RPR titers in primary syphilis, whereas in secondary syphilis *T. pallidum* DNA was more commonly detected in patients with higher serum RPR titers. Regrettably, the difference in the detection rates did not reach statistical significance. As serum RPR titers were relatively low in primary syphilis, only 9 patients with primary syphilis were recruited in this study with blood RPR titers at or beyond 128. On the contrary, serum RPR titers were higher in secondary syphilis and there were only 8 patients for whom serum RPR titers ranged from 0 to 16. The fact that only a few patients in these groups affected the statistics. This finding may therefore need more samples to be verified in further studies. RPR measures nontreponemal antibodies to lipoidal antigens, principally cardiolipin, released from damaged host cells and *T. pallidum*^21,22^. Therefore, RPR titers tend to correlate with disease activity and the *T. pallidum* burden. Our results suggest that when *T. pallidum* begins to disseminate into the blood from a local infection site, the small number of *T. pallidum* is not able to provoke enough humoral and cellular immune responses to clear pathogens at lower blood RPR titers for primary syphilis. On the contrary, secondary syphilis was observed at the stage of spirochetemia. *T. pallidum* may provoke an intense immune response to destroy the bacteria and, at the same time, the response may damage host cells, exposing the host to a greater concentration of lipoidal antigens and consequently increased titers of RPR. In the samples from latent syphilis, we detected *T. pallidum* DNA in only six patients with a serum RPR titer ≥ 64. Serum RPR titers were generally higher during early syphilis than during later stages of infection^23^. Thus, the six patients might be with early latent syphilis.

Our findings, together with those of previous studies^13, 24,25^ showed that nested PCR can be used to detect *T. pallidum* DNA in the peripheral blood of patients with syphilis at various stages. We further analyzed the relationship between blood RPR titers and the detection of *T. pallidum* DNA, and found that blood RPR titers were correlated with the blood *T. pallidum* burden. Importantly, these correlations were different in primary and secondary syphilis. This method is useful for detecting *T. pallidum* DNA for suspected cases of primary syphilis, especially when clinical symptoms are atypical or in circumstances when *T. pallidum* cannot be found in the chancre, and blood RPR and TPPA are non-reactive. In these cases, the detection of blood *T. pallidum* DNA can avoid or delay misdiagnosis. Our results indicate that the blood PCR method can be used to complement other methods to enhance the diagnosis of syphilis.

## Materials and methods

### Ethics statement and subjects

The study was approved by the Ethics Committee of the Shanghai Skin Disease Hospital. Between September 2015 and April 2017, eligible patients who sought medical care in the Sexually Transmitted Disease Clinic of the Shanghai Skin Disease Hospital were asked to participate in this study. Participants were interviewed with a brief questionnaire to collect medical and social-demographic information. Patients with therapeutic interventions were excluded. The diagnosis of the clinical stage of syphilis was determined based on a combination of a compatible history, clinical manifestations and the results of nontreponemal and treponemal tests in the serum. All eligible patients who agreed to participate in the study provided their written informed consent.

### Diagnostic criteria for primary, secondary, and latent syphilis

Primary syphilis was classified as having clinical manifestation of chancres or ulcers and one of the following laboratory confirmations: (i) positivity for spirochetes by dark-field microscopic examination; (ii) positive RPR confirmed by TPPA; or (iii) a negative result for spirochetes by dark-field microscopic examination and initially negative results of RPR and TPPA serologically becoming positive in the follow-up period (a maximum of 3 months).

Secondary syphilis was classified by (i) a positive RPR confirmed by TPPA and (ii) skin or mucocutaneous lesions.

Latent syphilis was defined by positive RPR confirmed by TPPA but without skin or mucocutaneous lesions, or any symptoms of syphilis; early latent syphilis was classified as an infection by *T. pallidum* within 2 years; and late latent syphilis was thereafter classified as having an infection after 2 years.

### PCR detection of *T. pallidum* in the blood

DNA was extracted from 1 ml of whole blood with anti-coagulant EDTA using the QiaAmp DNA mini blood Kit (Qiagen, Inc., Valencia, CA, USA) according to the manufacturer’s instructions. Nested PCR was performed initially with the outer primers of *polA* (sense: 5′-TGCGCGTGTGCGAATGGTGTGGTC-3′ and antisense: 5′-CACAGTGCTCAAAAACGCCTGCACG-3′). In a total volume of 50 μl, each reaction consisted of 10 μl of DNA, 25 μl of 2 × PCR master mix (Promega), and 0.6 μM primers. The PCR reaction was performed in an ABI 9700 thermocycler (Applied Biosystems, Foster City, CA) under the following cycling conditions: 94 °C for 4 min, 25 cycles at 94 °C for 1 min, 65 °C for 30 s, and 72 °C for 45 s, followed by 72 °C for 7 min. In the second round of PCR, 3 μl of the first reaction product was used in a total volume of 25 μl of reaction mixture with the sense primer (5′-GGATTGCATCCGCACGATAC-3′) and the antisense primer (5′-CAGCAGATGCAGATACCCCA-3′). This PCR reaction contained 12.5 μl of 2 × PCR master mix (Promega) and 0.6 μM primers. The cycling conditions consisted of 4 min at 94 °C then 30 cycles of 94 °C for 1 min, 60 °C for 30 s, and 70 °C for 30 s, followed by 72 °C for 7 min.

For the nested PCR amplifying the *Tpp47* gene, the outer primers included 5′-GTCAGCCTGTAGTATCCCG-3′ (sense) and 5′-TTCTGCACGTAAGGTAAGC-3′ (antisense). The inner primer sequences were 5′-TGCCATAACTCGCCATCAGA-3′ (sense) and 5′-CAACACGGTCCGCTACGACTA-3′ (antisense). The reaction mix and the cycling conditions were similar to those of *polA*. Appropriate positive (Nichols strain of *T. pallidum*) and negative (distilled water) controls were used with each set of reactions.

Single-step PCR was performed to amplify the *Tpp47* gene and the PCR reaction contained 12.5 μl of 2 × PCR master mix (Promega), 0.6 μM primers, and 10 μl of DNA. The inner primers of *Tpp47* were used. The cycling conditions were similar to those of the second round of PCR of *Tpp47*. For single-step PCR amplifying the polA gene, the PCR reaction contained 12.5 μl of 2 × PCR master mix (Promega), 0.6 μM primers, and 10 μl of DNA. The inner primers of *polA* were used. The cycling conditions were similar to those of the second round of PCR of* polA*.

Samples were kept at 4 °C until analysis by gel electrophoresis. The PCR products were electrophoresed on a 2% agarose gel together with a 100 bp DNA ladder (Takara) at 100 V for 20 min. The gels were visualized after staining with ethidium bromide.

### Statistical analysis

All statistical analyses were performed using SPSS software, version 16.0 (SPSS Inc., Chicago, IL, USA). Descriptive statistics were used to calculate the median and interquartile range. In addition, the *χ*^2^-test and the Fisher’s exact test were performed to compare the proportion between the groups. Differences were considered to be statistically significant at two-sided *P*-values < 0.05.

## Electronic supplementary material

Supplementary Figure 1

Supplementary Figure 1 legend
