# Correction

**DOI:** 10.1080/22221751.2020.1777775

**Published:** 2020-06-03

**Article title:** Sensitive detection of *Treponema pallidum* DNA from the whole blood of patients with syphilis by the nested PCR assay

**Authors:** Wang, C., Cheng, Y., Liu, B., Wang, Y., Gong, W., Qian, Y., Guan, Z., Lu, H., Gu, X., Shi, M., & Zhou, P.

**Journal:** Emerging Microbes & Infections

**DOI:** https://doi.org/10.1038/s41426-018-0085-2

This article was originally published with error in acknowledgement section. The correct version of the acknowledgement section is shown below:

This work was supported by the National Natural Science Foundation of China [81371862 and 81572039], the Basic Research Project of Shanghai Science and Technology Commission [15JC1403000], the Shanghai Natural Science Foundation [15ZR1437000, 16411961300, 17DZ2293300], Clinical Research Plan of SHDC [16CR1029B], and National megaproject on key infectious diseases [2017ZX10202102-001–007].

