# Supplementary figures and images for: Sensitive detection of Treponema pallidum DNA from the whole blood of patients with syphilis by the nested PCR assay
Source: Emerg Microbes Infect. 2018 May 9;7:83. doi: 10.1038/s41426-018-0085-2 (PMC5940865; doi:10.1038/s41426-018-0085-2)

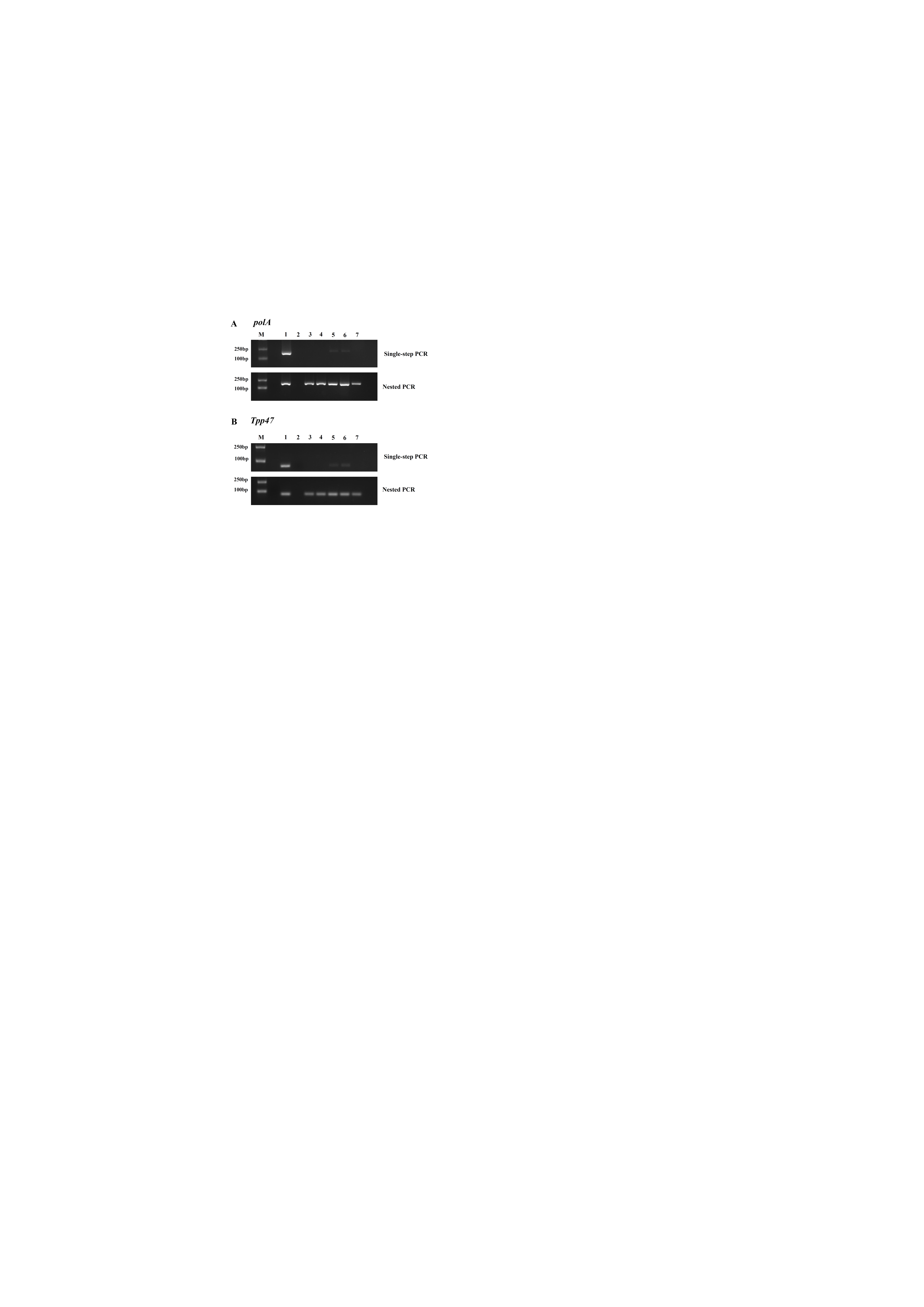

Supplement: Supplementary file 1 — Supplementary Figure 1 [file 41426_2018_85_MOESM1_ESM.tif]
