# Supplementary material for: Sensitive detection of Treponema pallidum DNA from the whole blood of patients with syphilis by the nested PCR assay
Source: Emerg Microbes Infect. 2018 May 9;7:83. doi: 10.1038/s41426-018-0085-2 (PMC5940865; doi:10.1038/s41426-018-0085-2)
Supplement: Supplementary file 2 — Supplementary Figure 1 legend [file 41426_2018_85_MOESM2_ESM.docx]

***Figure Legend***

**Figure S1**

**Amplicons of Tpp47 and polA from samples using single-step PCR and nested PCR.**

(A) Amplicons of polA from samples using single-step PCR and nested PCR. M, Marker; lane1, positive control; lane2, negative control; lane 3,4,5,6, and 7, samples; (B) Amplicons of Tpp47 from samples using single-step PCR and nested PCR. M, Marker; lane1, positive control; lane2, negative control; lane 3,4,5,6, and 7, samples. The five samples were Tpp47 and polA positive verified by nested PCR.
